# Engineering allogeneic type 1 regulatory T cells: a scalable, off-the-shelf platform for restoring immune tolerance

**DOI:** 10.3389/fimmu.2026.1848770

**Published:** 2026-07-09

**Authors:** Molly Javier Uyeda, Audrey Re, Chunyi Tang, Melissa McLaughlin, Daryl Humes, Steven Sexton, Robert Freeborn, Isaura Villalba, Kevin Nugent, James Adams, Jie Wei, Matt Breton, Xavier Paliard, Maximilian Richter, Maria Grazia Roncarolo, Ryan Bjordahl

**Affiliations:** Tr1X Inc., San Diego, CA, United States

**Keywords:** allogeneic, autoimmunity, cell therapy, regulatory, tolerance, Tr1, IL-10

## Abstract

Type 1 regulatory T (Tr1) cells are IL-10–producing CD4^+^ regulatory T cells that play a central role in maintaining peripheral immune tolerance. Prior investigations of autologous Tr1 cell-based therapies have demonstrated clinical promise in graft-versus-host disease (GvHD), but widespread clinical application has been constrained by challenges in scaling cell production. TRX103 is an engineered, allogeneic Tr1 cell therapy manufactured using a fully compliant, current Good Manufacturing Practice-compatible process. TRX103 is generated by pooling engineered CD4^+^ T cells from three healthy donors that have been transduced with a lentiviral vector encoding human IL-10 and a truncated human nerve growth factor receptor, CD271. *In vitro*, off-the-shelf TRX103 suppressed effector T cell proliferation and inhibited pro-inflammatory cytokine production by monocytes from both healthy donors and patients with Crohn’s disease. *In vivo*, TRX103 homed to intestinal tissues and prevented disease development in a xenogeneic GvHD model. Phenotypic and functional analyses of TRX103 revealed the acquisition of a distinct cellular identity characterized by reduced expression of activation and immune synapse–associated molecules and minimal allogeneic stimulatory capacity. Together, these findings define a distinct immunomodulatory profile for TRX103 and support its clinical development as a scalable allogeneic cell therapy for immune-mediated diseases. TRX103 is currently being evaluated in a Phase 1/2a study in treatment-refractory Crohn’s disease (NCT06721962) and a Phase I study for GvHD prevention in hematopoietic stem cell transplantation recipients (NCT06462365).

## 1 Introduction

Type 1 regulatory T (Tr1) cells are a subset of CD4^+^ regulatory T (Treg) cells characterized by the secretion of high amounts of IL-10 that play a central role in maintaining peripheral immune tolerance and controlling inflammatory immune responses (1). FOXP3^-^ Tr1 and FOXP3^+^ Tregs perform complementary roles and work together to maintain immune tolerance to self-antigens and non-pathogenic foreign antigens (2). Unlike FOXP3^+^ Tregs, a single master transcription factor driving Tr1 Treg cellular identity and regulatory function has not been identified. Tr1 Treg cell identity and function appear to be driven by a network of transcription factors that work in concert to induce immunoregulatory function and a unique cytokine secretion profile consisting of high levels of IL-10 and low levels of IL-4 (3).

The IL-10-driven regulatory program of Tr1 Tregs is particularly important in achieving and maintaining peripheral immune tolerance to non-pathogenic antigens. Tr1 Tregs specific for host alloantigens were first identified in a patient with severe combined immunodeficiency who developed sustained mixed chimerism after successful treatment with a mismatched allogeneic fetal liver and thymus transplant (4, 5). Extensive preclinical studies aimed at elucidating the mechanisms underlying Tr1 Treg cell development and immunoregulatory function followed this early clinical observation. In T cell transfer–based mouse models of inflammatory bowel disease, in which adoptive transfer of pathogenic T cells induces disease, Tr1 Tregs conferred protection from colitogenic inflammation in an IL-10-dependent manner (6, 7). IL-10 mediates these effects by inhibiting effector T-cell proliferation, suppressing pro-inflammatory cytokine production by myeloid cells, and downregulating antigen presentation through reduced expression of HLA-Class II, CD80, and CD86 (3, 8).

The indispensable role of IL-10 signaling in maintaining immune homeostasis is further illustrated by patients harboring loss-of-function mutations in genes encoding IL-10 or the IL-10 receptor. A systematic review encompassing individuals with IL-10 or IL-10 receptor mutations found that all patients presented with gastrointestinal disease, frequently manifesting as Crohn’s disease or colitis (9). Gastrointestinal disease is thought to result from sustained activation of mononuclear immune cells in response to bacterial components, leading to excessive production of inflammatory cytokines such as TNF-α and consequent intestinal mucosal injury (10). A mouse model of inflammatory bowel disease utilizing a dominant-negative IL-10 receptor showed that the IL-10 receptor was required for sustained Tr1 Treg cell functionality, and patients with inflammatory bowel disease such as Crohn’s disease and ulcerative colitis had a defect in IL-10-producing FOXP3^−^ CD4^+^ T cells (11). Despite the abundance of evidence illustrating the central role of IL-10 and IL-10-secreting Tr1 Tregs in preventing autoimmune and inflammatory conditions, systemic administration of recombinant IL-10 as a therapeutic has had limited clinical benefit in diseases such as psoriasis and inflammatory bowel disease (12, 13). This could be due to a short serum half-life, insufficient tissue localization, and dose-limiting systemic effects (14). These findings suggest the importance of spatially and temporally controlled IL-10 delivery within inflamed tissues — an attribute a Tr1 Treg-based cell therapy could provide.

Early clinical studies explored antigen-specific Tr1 Treg cell products from a variety of sources, including food antigen-specific Tr1 Treg cell clones (15, 16) and donor-derived, alloantigen-specific Tr1 Treg-enriched products evaluated in the setting of HSCT (17, 18). In graft-versus-host disease (GvHD), where immune dysregulation arises following HSCT, a proof-of-concept clinical trial demonstrated that a single infusion of IL-10-anergized donor T cells enriched in Tr1 Tregs prevented both acute and chronic GvHD in a proportion of patients with hematological malignancies (17). More recently, an alloantigen-specific Tr1 Treg-enriched cell product demonstrated long-term persistence of donor-derived Tr1 Tregs alongside clinical benefit in a similar setting (18, 19). Although these proof-of-concept studies demonstrated clinical promise, they have been constrained by limited feasibility, largely due to their reliance on autologous or donor-derived, antigen-dependent cell products that are challenging to standardize and scale.

A key advancement by Andolfi et al. engineered polyclonal CD4^+^ T cells by transduction with a bidirectional lentiviral vector (LVV) encoding *IL-10* and a truncated nerve growth factor receptor selection marker (NGFR/CD271). Using this strategy, a homogeneous population of CD4^+^ IL-10–expressing T cells with Tr1 Treg-like functional properties could be generated from abundant donor starting material (20–22). While this work established the foundation for generating engineered Tr1 Treg-like cells and demonstrating the biology of T cell-based delivery of IL-10, key challenges related to scalability and clinical-grade manufacturing remained.

To address these unmet translational challenges, we developed TRX103, an engineered allogeneic Tr1 Treg cell therapy designed as a standardized, off-the-shelf product capable of delivering IL-10 in a spatiotemporally controlled manner. TRX103 incorporates a defined multi-donor pooling strategy to mitigate donor-specific variability using a GMP-compatible process. TRX103 has a unique cellular phenotype, which includes secretion of high levels of IL-10 and downregulation of molecules associated with antigen presentation and allogeneic T-cell recognition. TRX103 cells display immunosuppressive and anti-inflammatory activity *in vitro* against both T cells and monocytes and prevent disease in a xenogeneic GvHD model *in vivo*. These findings have direct translational relevance, as TRX103 is currently being evaluated in two first-in-human clinical trials: the RESTORE Phase 1/2a study in patients with treatment-refractory Crohn’s disease (NCT06721962) and a Phase I study for prevention of GvHD in HSCT recipients with hematologic malignancies (NCT06462365).

## 2 Materials and methods

### 2.1 Generation of TRX103

CD4^+^ T cells were isolated from Leukopaks or Leukocyte Reduction System Chambers (LRSC) using CD4^+^ enrichment kits from Miltenyi Biotec (clinical-scale production; Gaithersburg, MD, USA) or Stem Cell Technologies (research-scale production; Stem Cell Technologies, Kent, WA, USA). Cell generation is accomplished using G-Rex culture vessels (Wilson Wolf, Minneapolis, MN, USA) at the research-scale or using the CliniMACS Prodigy for clinical-scale, GMP grade material. TexMACS media (Miltenyi Biotec) supplemented with human serum was used for TRX103 productions. Isolated CD4^+^ T cells from 3 different donors were activated with T Cell TransACT (Miltenyi Biotec) and transduced with LVV encoding full-length *IL10* and truncated *NGFR/CD271* complementary DNA (cDNA) (22). Transduced cells were enriched with CD271 microbeads (Miltenyi Biotec), reseeded and continuously replenished with fresh medium over the course of the production. On day of harvest, the cells were phenotyped for CD4 and CD271 co-expression and pooled at a 1:1:1 ratio based on the percentage of viable CD4^+^CD271^+^ T cells. Non-IL-10 overexpressing controls were generated in parallel with TRX103. CD4^+^ T cells were transduced with a LVV encoding an IL-10 cDNA with multiple mutations (start codons removed and premature stop codons added) that prevent the translation of IL-10 protein and are referred to as CD4^ΔIL-10^.

### 2.2 Flow cytometry

Cell surface staining was performed at room temperature for 15 minutes followed by multiple washes. For intracellular staining, the cells were fixed, permeabilized, and stained with the CytoFix/CytoPerm kit according to the manufacturer’s recommendations (BD Biosciences, Franklin Lakes, NJ, USA). For phenotyping of circulating cells in mice, peripheral blood was collected in K2EDTA tubes. Blood was incubated directly with antibodies followed by lysing and fixing red blood cells (RBC Lysis/Fixation Solution (10X), Biolegend, San Diego, CA, USA). Precision Count Beads (Biolegend) were spiked into each sample before acquisition to obtain cell counts. Unbiased cell-surface characterization was performed using LEGENDScreen Human Cell PE Kit (Biolegend) following the manufacturer’s recommendation. Flow cytometric samples were acquired on an Attune NxT flow cytometer (Thermo Fisher Scientific, Waltham, MA, USA) and FCS files were analyzed using FlowJo 10 (BD Biosciences). All antibodies and reagents utilized in flow cytometry are detailed in **Supplementary Table 1**. IL-10 expression in TRX103 cells was detected on cells cultured in complete media (TexMACS media [Miltenyi Biotec] with 3% AB male serum; unstimulated) or complete media plus CD3/CD28/CD2 Immunocult (stimulated; Stem Cell Technologies) for 72 hours. Afterwards, cells were stained using an on-cell IL-10 Secretion Assay following manufacturer’s recommendations (Miltenyi Biotec) followed by surface marker staining.

### 2.3 Soluble protein quantification

TRX103 cells were thawed and cultured in complete media (unstimulated) or in the presence of CD3/CD28/CD2 Immunocult (stimulated; Stem Cell Technologies) for 48 hours and cell supernatants were frozen and analyzed with an DuoSet ELISA for IL-10 (R&D Systems, Minneapolis, MN, USA). Cytokine quantification on cell supernatants in mixed lymphocyte reactions (MLR) was performed using Mesoscale Discovery U-PLEX for IL-10, IFN-γ, and TNF-α (MSD, Rockville, MD, USA).

### 2.4 T cell proliferation assay

Previously isolated T cells were thawed and rested for 4 hours at 37 °C. Dead cells were removed with the EasySep™ Dead Cell Removal (Annexin V) Kit (Stem Cell Technologies) and then labeled with CellTrace Violet (CTV; Thermo Fisher Scientific). Cells were plated at 50,000 cells/well in 96-well U-bottom plates in complete media consisting of TexMACS media (Miltenyi Biotec) supplemented with 3% male AB serum. Cells were activated with Human T-Activator CD3/CD28 Dynabeads (Thermo Fisher Scientific) at a 1:10 bead-to-cell ratio and cultured at 37 °C with 5% CO_2_. Proliferation was assessed by flow cytometry 72 hours after activation, with CTV dilution used as a readout of cellular division.

### 2.5 Myeloid cell stimulation assays

Conditioned media (CM) was generated by stimulating TRX103 or controls in complete media with CD3/CD28/CD2 Immunocult (Stem Cell Technologies) for 48 hours and cell supernatants were frozen. Peripheral blood mononuclear cells (PBMC) were isolated from LRSC (Versiti, Milwaukee, WI, USA) using Ficoll-Paque density gradient centrifugation (Cytiva, Marlborough, MA, USA).

Inhibition of TNF-α production by stimulated PBMCs was assessed using PBMC isolated from LRSC or Leukopaks by gradient centrifugation using Ficoll-Paque prior to experiment setup. Frozen PBMC were thawed and plated in RPMI and 10% (v/v) FBS in a 96-well U-bottom plate at 1E6 cells/well. PBMC were rested at 37 °C. After 2 hours, all indicated reagents were added simultaneously. For experiments using healthy donors, 50 ng/mL lipopolysaccharide R515 (Enzo Life Sciences, Farmingdale, NY, USA) was added to activate monocytes, and for experiments using PBMC from patients with Crohn’s disease, 50 ng/mL LPS and 33 ng/mL rh-IFN-γ (R&D Systems) was added to activate monocytes. When called for, CM was used at 12.5% volume/volume (v/v), and 1 ng/mL of rhIL-10 was used. Cells were incubated for 24 hours. Supernatant were collected and frozen at -80 °C. Quantification of TNF-α was measured by MSD following the manufacturer’s instructions.

Inhibition of NLRP3 inflammasome activation in monocytes was evaluated for healthy donors and Crohn’s disease patients. For assays using healthy donor material, monocytes were enriched from LRSC via magnetic depletion using the Human Monocyte Isolation Kit (Stem Cell Technologies) following manufacturer’s instructions. Enriched monocytes were plated in RPMI medium (Gibco, Grand Island, NY, USA) at 1.5E5 cells/well in a 96-well flat bottom plate, and 10 μM MCC950 (Invivogen, San Diego, CA, USA) was added to control wells. After 1 hour, 1 μg/mL of LPS (Enzo Life Sciences), conditioned media (25% v/v), and 10 ng/mL recombinant human (rh) IL-10 were added. After 5.5 hours, 10 μM of Nigericin (Sigma-Aldrich, St. Louis, MO, USA) was added. At 6 hours, supernatant was collected and frozen at -80 °C. Quantification of IL-1β was measured using Human IL-1β/IL-1F2 DuoSet ELISA (R&D Systems) or MSD U-Plex following the manufacturer’s instructions. For assays using Crohn’s disease donor material, frozen PBMC were utilized and no monocyte enrichment was performed. All steps were conserved as described for healthy donor material except 50 ng/mL of LPS was incubated for 23.5 hours before the addition of Nigericin for the last 30 minutes of the 24-hour assay.

Human blood samples from patients with Crohn’s disease were obtained from commercial vendors (Sanguine Bio, Los Angeles, CA, USA; Precision for Medicine, Bethesda, MD, USA). As samples were de-identified and obtained through commercial vendors with existing IRB oversight, this study was determined to be exempt from additional institutional review.

### 2.6 T cell suppression assay

Mature dendritic cells (matDC) were generated by isolating CD14^+^ monocytes from PBMC and culturing them using the ImmunoCult™ Dendritic Cell Culture Kit (Stem Cell Technologies). Allogeneic CD3^+^ T cells were isolated from PBMC using the EasySep™ Human T Cell Isolation Kit (Stem Cell Technologies). Isolated T cells and matDC were cryopreserved until use and recovered on the day of the assay. Allogeneic CD3^+^ T cells were labeled using Cell Trace Violet (CTV) proliferation dye (Thermo Fisher Scientific). The final co-culture was prepared with TRX103, CTV-labeled responder T cells, and matDC at a 10:10:1 ratio (TRX103: Responders: matDC) in the presence of 50 ng/mL soluble anti-CD3 monoclonal antibody (Clone OKT3, Miltenyi Biotec). After four days of co-culture, samples were stained and acquired on an Attune NxT flow cytometer. Suppression of proliferation was calculated as (%Proliferation of Responders alone – %Proliferation of Responders with Suppressors)/%Proliferation of Responder alone.

### 2.7 Mixed lymphocyte reaction

For evaluation of activation-induced markers on responder T cells, cryopreserved TRX103 cells were thawed and plated at 1E6 cells/well in a 96 well U bottom plate. Allogeneic PBMC were thawed and plated at a 1:1 ratio with TRX103. After 4 hours of incubation at 37 °C, Golgi Plug and Golgi Stop were added according to the manufacturer’s recommendation (BD Biosciences). The co-cultures were incubated for an additional 20 hours at 37 °C before being harvested, stained for surface and intracellular markers, and acquired on an Attune NxT flow cytometer (Thermo Fisher Scientific).

Cryopreserved allogeneic PBMC were thawed and labeled using CTV proliferation dye (Thermo Fisher Scientific). TRX103 were thawed and labeled using Carboxyfluorescein succinimidyl ester (CFSE) proliferation dye (Thermo Fisher Scientific). CFSE-labeled TRX103 and CTV-labeled responder PBMC were co-cultured at a 1:1 ratio. After 4 days of co-culture, cell supernatants were collected, and complete media was replaced. After 7 or 8 days of co-culture, samples were evaluated for additional surface marker expression including CD8, CD4, CD3, and CD56 to allow for more precise sub-gating and to count live cells that were acquired on an Attune NxT flow cytometer (Thermo Fisher Scientific).

### 2.8 *In-vivo* xenogeneic (xeno) graft versus host disease

#### 2.8.1 PBMC induction of xeno-GvHD

Six- to eight-week-old female NOD.Cg-*Prkdc^scid^ Il2rg^tm1Wjl^*/SzJ (NSG) mice were purchased from the Jackson Laboratory (Bar Harbor, ME, USA). On Study Day -1, female NSG mice were subject to total body irradiation (TBI) (175 cGy for animals with body weight below 20 g and 200 cGy for animals with body weight above 20 g). On Study Day 0, animals were infused intravenously (IV) with a combination of cells (TRX103/PBMC) at the indicated amount or vehicle (PBS) with 5 mice per group. xeno-GvHD was induced by the administration of 5E6 human PBMC. xeno-GvHD scoring is described in **Supplementary Table 2**. Mice were determined to have xeno-GvHD when any one of the following criteria was met: mice with two consecutive combined xeno-GvHD scores ≥5; mice with a combined xeno-GvHD score of ≥4 that were found dead at the next assessment; mice with a combined xeno-GvHD score ≥6; mice with a combined xeno-GvHD score of ≥7 and/or weight loss of ≥30% of starting weight, which also triggered euthanasia for humane reasons. All surviving animals at the end of the study, Study Day 58, were euthanized by isoflurane overdose followed by cervical dislocation after GvHD scoring was completed. Lymphocyte counts from peripheral blood were calculated as followed: (number of cells obtained by flow cytometry × number of beads added to the tube)/number of beads obtained by flow cytometry. Cell counts per μL of whole blood were calculated by dividing the lymphocyte counts by the volume of blood sampled. The gating strategy is shown in **Supplementary Figure 6A**.

#### 2.8.2 CD4^+^ T cell induction of xeno-GvHD

On Day -3, all animals received sublethal TBI as in the PBMC model. After 72 hours, animals were divided into 4 groups (n = 10 mice/group) and injected with the following: vehicle (PBS), CD4^+^ T cells (5E6 cells/animal in PBS), CD4^+^ T cells and TRX103 cells at a 1:1 ratio (5E6 CD4^+^ T cells plus 5E6 TRX103 cells/animal in PBS), and TRX103 cells (5E6 cells/animal in PBS). Animals were monitored for visible signs of xeno-GvHD and body weight twice weekly through Day 7 and then daily from Day 8 until the end of the study (Day 16 or 17). Half of the animals were euthanized on Day 16, with the remaining animals euthanized by CO2 inhalation and exsanguination from abdominal vena cava on Day 17 for tissue collection and histopathology analysis.

#### 2.8.3 Histology

All tissues were fixed in 10% neutral buffered formalin and processed to wax blocks. Formalin-fixed paraffin-embedded tissue sections (4 μm) were placed on positively charged TOMO slides (cat# TOM-1190), dried, and baked at 60 °C for 1 hour to adhere tissue, then deparaffinized using three cycles of Pro-Par clearant dipping (20×) and submersion (10 minutes). Slides underwent rehydration in reagent alcohol: twice in 100% (20× dips, 1.5 minutes submersion) and once in 90% (20× dips, 1.5 minutes submersion), followed by a 2-minute DI water rinse. Antigen retrieval was performed in pH 6.0 citrate buffer in a Biocare Medical Decloaking Chamber NxGen (Program 5: 110 °C, 15 min; Biocare Medical, Pacheco, CA, USA) and then cooled to room temperature. Autofluorescence was quenched in PBS with 2.4 mM NaOH and 1.47 M H2O2 solution under LED for two 45-minute cycles. The solution was replaced between cycles. The slides were washed twice with PBS for 5 minutes and then placed in a Freequenza rack, blocked with 5% normal donkey serum and 0.3% Triton X-100 in PBS for 1 hour, and immunostained with rabbit anti-human CD4 antibody conjugated to Alexa Fluor 555 (Abcam Cat# ab280849, RRID: AB_3107080, clone EPR6855, 2.5 μg/mL in PBS; Waltham, MA, USA). Following overnight incubation at 4 °C, slides were warmed up to room temperature for 1 hour, washed 4 times with PBS, stained with Hoechst 33342 (Invitrogen, cat# H3570, 10 μg/mL in PBS), washed 5 times with PBS, and embedded with Prolong Gold under #1.5 coverslip. Slides were dried and digitized with ZEISS AxioScan.Z1 slide scanner using 20x 0.8NA objective, Colibri 7 LED light source, single-band filter sets for capturing DNA signal, tissue autofluorescence and Alexa Fluor 555 antibody using Hamamatsu OrcaFlash4.0 V2 camera. Image analysis was done in QuPath software (23).

### 2.9 TCR sequencing

1E6 cells (TRX103 and CD4^IL-10^ sublots) were pelleted and frozen at -80 °C. Genomic DNA was isolated from the cell pellets following the manufacturer’s instructions in the DNeasy Blood & Tissue Kit (Qiagen). Genomic DNA from all tested lots and sublots was shipped to CD Genomics for further processing (CD Genomics, Shirley, NY, USA). All downstream processing such as DNA quality control, library generation, sequencing, and data analysis was performed by CD Genomics.

### 2.10 Generative AI

Claude for Mac Co-work (Claude 1.1.8629) was used to edit the text for grammar, readability, and clarity.

### 2.11 Statistics

All statistical analyses were performed using GraphPad Prism version 10 (GraphPad Software, Boston, MA, USA). Statistical significance was defined as a false discovery rate (FDR)–adjusted *p* value (padj) < 0.05. Comparisons between two groups were performed using paired t-tests and the Mann–Whitney U test applied to unpaired samples. For comparisons involving more than two groups, statistical significance was assessed using two-way analysis of variance (ANOVA) followed by Tukey’s or Bonferroni’s *post hoc* multiple-comparison correction, as indicated. Bars and error bars represent mean and standard deviation (SD) unless otherwise noted. *p<0.05, **p<0.01, ***p<0.001, ****p<0.0001.

## 3 Results

### 3.1 Generation of engineered Tr1 Tregs using a pooled-product strategy

**Figure 1:**
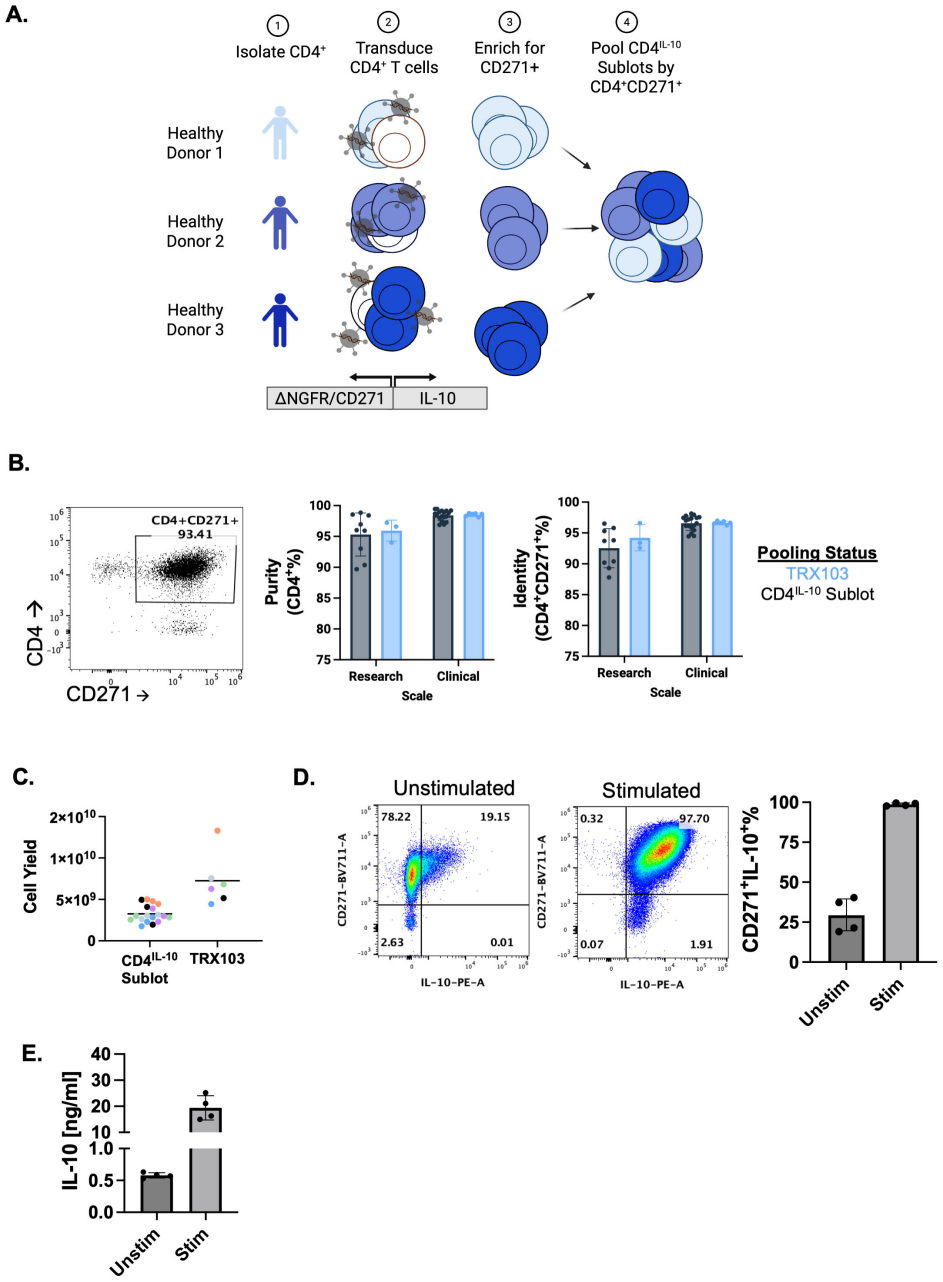
Generation of a Tr1 Treg-cell based adoptive cell therapy. **(A)** Schematic of the TRX103 production process. Three unrelated donors' CD4^+^ T cells are transduced, enriched for CD271^+^, and pooled in an even ratio (1:1:1) normalized by CD4^+^CD271^+^%. **(B)** Representative flow cytometry analysis of TRX103 lot Final Product by CD271 and CD4. Summary of CD4^+^ T cell purity and CD4^+^CD271^+^ identity in CD4^IL-10^ sublots (black, n = 9 research-scale and n = 18 clinical-scale lots) and the pooled TRX103 final product (blue, n = 3 research-scale and n = 6 clinical-scale lots). **(C)** Total viable cell yield from each CD4^IL-10^ sublots (n = 18) and TRX103 (shared colors indicate each TRX103 pooled product, n = 6, and its three constituent donor sublots). **(D)** Representative flow cytometry analysis of IL-10 and CD271 expression in TRX103 unstimulated with media only or stimulated with anti-CD3/CD28/CD2. Summarized CD271^+^IL-10^+^ % in clinical lots (n = 4). **(E)** Secreted IL-10 [ng/mL] in cell culture supernatants containing TRX103 cells (n = 4). Unstim= unstimulated with media only, Stim = stimulated with anti-CD3/CD28/CD2. Created in BioRender. Uyeda, M. (2026) https://BioRender.com/m6zhw96.

TRX103 cells are manufactured from cryopreserved CD4^+^ T cells isolated from healthy donors. Manufacturing consists of three major process events: transduction of CD4^+^ T cells with a lentiviral vector encoding *IL10* and truncated *NGFR/CD271* transgenes, enrichment of CD271^+^-transduced cells, and pooling of three manufactured sublots, designated CD4^IL-10^ cells, at a 1:1:1 ratio to formulate the final TRX103 drug product (**Figure 1A**). The process yields a highly pure product at both research- and clinical-scale manufacturing, with greater than 95% purity for CD4^+^ T cells (98.5 ± 0.3%, n = 6), and transduced CD4^+^CD271^+^ cells (96.6 ± 0.3%, n = 6) at clinical-scale manufacturing (**Figure 1B**). The pooling of three donors enables higher yields per clinical lot, yielding over 7E9 cells (n = 6) on average (**Figure 1C**). Additionally, pooling increases the T cell receptor (TCR) repertoire richness as the number of unique clonotypes from each donor are additive and increases the TCR repertoire evenness by diluting the frequency of any expanded, dominant clones (**Supplementary Figure 1**). In one clinical-scale manufacturing run, the three most abundant clonotypes present in one CD4^IL-10^ sublot, V5-J22 (11.25%), V5-J11 (6.71%), and V24-J23 (6.65%), were reduced to 7.41%, 3.60%, and 3.45%, respectively, in the pooled TRX103 product, thereby reducing donor-specific clonal dominance in the final product (**Supplementary Figure 1**).

To validate elevated IL-10 secretion in TRX103 at both the single cell and population level, we measured secreted IL-10 levels by a flow cytometry-based IL-10 capture assay and separately by ELISA. The IL-10 capture assay detects IL-10 secretion by individual cells and specifically quantifies cytokine secretion as opposed to synthesis and has previously been used to isolate live Tr1 Tregs (24). Secreted IL-10 was detectable in a portion of TRX103 cells at rest (30.0 ± 11.0% CD271^+^IL10^+^, n = 4), while nearly all TRX103 cells were IL-10^+^ after stimulation (99.0 ± 0.9% CD271^+^IL10^+^, n = 4, **Figure 1D**). IL-10 production per cell was similarly increased after stimulation, as the IL-10 fluorescent signal in the capture assay positively shifted in IL-10^+^ TRX103 cells (IL-10 geometric mean fluorescent intensity 1,259 ± 74 unstimulated vs 10,094 ± 3,638 stimulated, n = 4). Soluble cytokine quantification of IL-10 in cell culture supernatants allowed us to assess IL-10 secretion at a population level. We observed modest IL-10 secretion at rest (0.6 ± 0.04 ng/mL, n = 4) and robust secretion after stimulation (19 ± 4.7 ng/mL, n = 4), which was not observed in control T cells (**Figure 1E**; **Supplementary Figure 2A**). After stimulation, TRX103 also produced significant levels of IFN-γ and IL-22, but low levels of IL-4 (**Supplementary Figure 2B**). This cytokine profile is similar to that previously reported for natural Tr1 Tregs (7, 24). Collectively, these findings establish that TRX103 is reproducibly manufactured at high yield and purity with a GMP-compliant process and that the final product preserves the defining Tr1 Treg cytokine profile.

### 3.2 IL-10 overexpression drives a unique immunomodulatory program in TRX103

**Figure 2:**
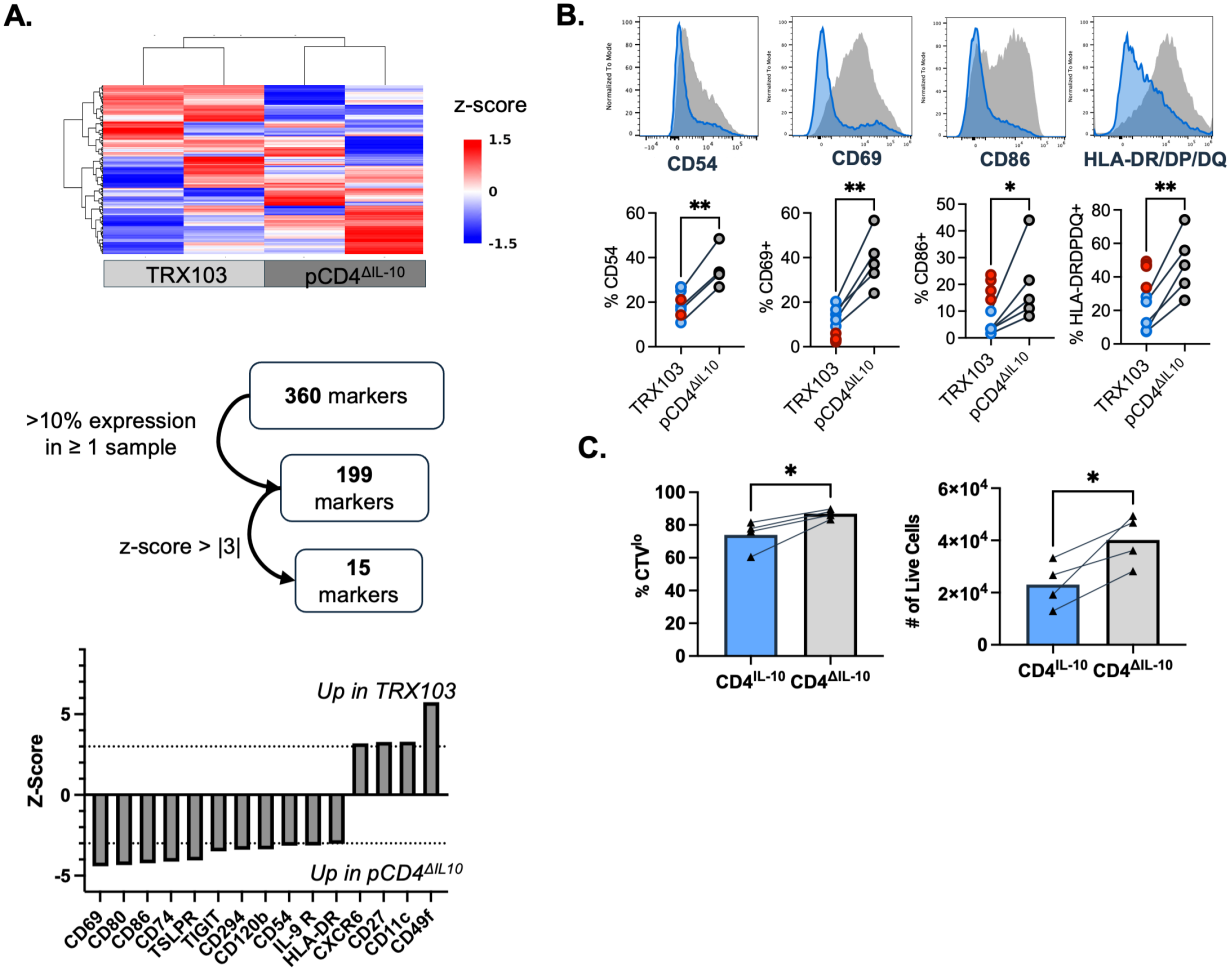
TRX103 manufacturing functionally reprograms CD4^+^ T cells. **(A)** Filtering strategy of 360 cell-surface markers quantified in LEGENDScreen to identify 15 markers differentially expressed by unstimulated research-scale TRX103 compared to pCD4^ΔIL-10^ controls from the same donors n = 2 matched pairs). Heatmap using Euclidean clustering of 199/360 surface markers that had at least 10% expression in ≥ 1 sample. Z-score of all 15 markers identified from filtering strategy. **(B)** Extended flow validation with example histograms and summarized data on additional TRX103 lots (blue: research-scale, n = 5; red clinical-scale, n = 4) and respective controls (gray, n = 5). **(C)** Frequency of CTV^lo^ cells, which indicate the number of cells that have divided at least once (left) and live cell counts (right) after polyclonal anti-CD3/CD28 stimulation for 72 hours (n = 4). CTV = Cell Trace Violet; pCD4^∆IL-10^, pooled non-IL-10 secreting control CD4^∆IL-10^; *p<0.05, **p<0.01.

To define the molecular and functional programs induced by IL-10 gene integration and protein overexpression, we performed flow cytometric and functional analyses on TRX103. Control sublots were generated in parallel with CD4^IL-10^ but were transduced with a LVV encoding a mutated IL-10 transgene that does not produce functional IL-10 protein (CD4^ΔIL-10^; see Materials & Methods). The same three donors pooled in TRX103 were also used to generate the pooled (p) control, pCD4^ΔIL-10^, allowing for the isolation of IL-10-driven effects from non-specific donor- or batch-specific effects. Given IL-10’s known ability to suppress antigen presenting cell (APC) function and induce anergy in T cells (20, 25), we hypothesized that IL-10 overexpression may also directly regulate T cell-surface marker expression through autocrine signaling. To evaluate this, we performed unbiased cell-surface profiling of 360 cell surface proteins. This analysis identified 15 highly-differentially expressed markers (z-score ≥ 3) that distinguish TRX103 from pCD4^ΔIL-10^ controls (**Figure 2A**). Four out of the 15 markers were upregulated, including CXCR6, CD27, CD11c, and CD49f; however, the majority were downregulated (**Figure 2A**) and included CD54, CD74, CD69, CD80, CD86, and HLA Class II molecules. Reduced cell-surface expression of these proteins was confirmed on additional TRX103 lots manufactured at both research (n = 5) and clinical scale (n = 4), with matched pCD4^ΔIL-10^ controls included where available (p<0.01 CD54, CD69, HLA-DR/DP/DQ; p <0.05 CD86; not significant CD74) (**Figure 2B**; **Supplementary Figure 3**).

### 3.3 TRX103 exhibits reduced proliferative capacity and attenuated allogeneic immunogenicity

To determine whether the reduced activation phenotype identified by cell-surface phenotyping also reflected a functional reduction in proliferative capacity, we measured proliferation of CD4^IL-10^ and CD4^ΔIL-10^ control cells following stimulation with anti-CD3/CD28 antibodies. Individual CD4^IL-10^ sublots were selected rather than the TRX103 lot to eliminate the masking effect inherent to averaging donor responses. Proliferation of CD4^IL-10^ sublots upon stimulation was significantly reduced compared to CD4^ΔIL-10^ control sublots generated from the same donors (**Figure 2C**; p<0.03). Both the frequency of CTV low (CTV^lo^) cells, which reflects the proportion of cells that have undergone at least one division, and total live cell counts, which captures the overall expansion of the culture regardless of the number of divisions, were lower in CD4^IL-10^ sublots relative to CD4^ΔIL-10^ controls. These data are consistent with the cell-surface analysis and suggests that expression of the IL-10 transgene dampens the activation of the engineered CD4^+^ T cells and reduces their proliferation capacity following stimulation through the TCR.

**Figure 3:**
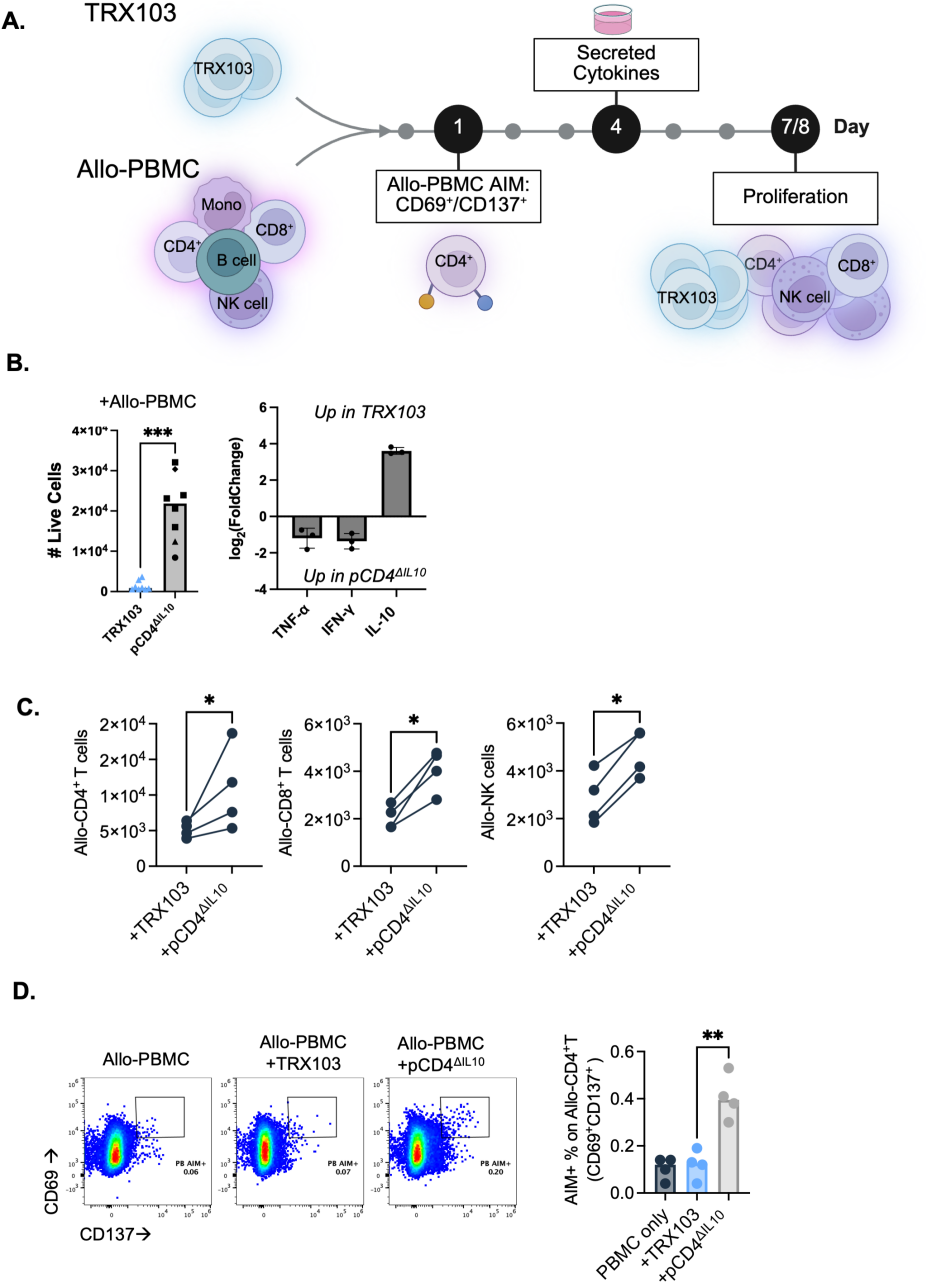
TRX103 cell reprogramming enables off-the-shelf delivery by modulating allogeneic-reactive responses. **(A)** Graphical representation of 2-way mixed lymphocyte reaction with TRX103 and allogeneic PBMC. **(B)** TRX103 or pCD4^ΔIL-10^ cells were co-cultured with allogeneic PBMC. Live cell counts are reported after 7–8 days (n = 8) and differential cytokines found in cell culture supernatants after 4 days (n = 3) of co-culture. **(C)** Live cell counts of the indicated allogeneic PBMC-derived immune subset (CD4^+^ T cells, CD8^+^ T cells, NK cells) after 7–8 days of co-culture with TRX103 or pCD4^ΔIL-10^ cells (n = 4). **(D)** Representative flow cytometry analysis and summarized data of activation-induced marker expression measured by CD137 and CD69 after 24-hour co-culture of TRX103 or pCD4^ΔIL-10^ with allogeneic-PBMC (n = 4). AIM, activation-induced markers; Allo, allogeneic; pCD4^∆IL-10^, pooled non-IL-10 secreting control CD4^∆IL-10^; *p<0.05, **p<0.01, ***p<0.001. Created in BioRender. De vries, D. (2026) https://BioRender.com/ tznynwr.

Although antigen presentation is not typically associated with CD4^+^ T cells, the presence of HLA-Class II and T cell costimulatory molecules are critical for allogeneic recognition and rejection by host T cells. The coordinated reduction in cell surface HLA-DR/DP/DQ, CD54, CD80, and CD86 would be expected to reduce the stimulatory capability of the TRX103 cells and therefore the likelihood of inducing an allogeneic T cell response. To test this hypothesis, we performed a series of two-way mixed lymphocyte reactions (MLR) with allogeneic PBMC (allo-PBMC) and TRX103 or pCD4^ΔIL-10^ control cells. By using CD271 as a marker for TRX103 and dye-based cell labeling, we were able to discriminate both cell populations to measure the allogeneic reactivity of TRX103 against allo-PBMC and, conversely, the stimulation of allo-PBMC by TRX103 (**Figure 3A**). As predicted, TRX103 expanded less than pCD4^ΔIL-10^ controls in the two-way MLR (p <0.001; **Figure 3B**), consistent with the reduced proliferation observed following anti-CD3/CD28 bead stimulation. However, the relative contributions of reduced proliferation versus increased cell death to the observed reduction in cell numbers were not independently assessed. Cell-culture supernatants contained reduced levels of TNF-α and IFN-γ but increased IL-10 in TRX103 cultures compared to pCD4^ΔIL-10^ (**Figure 3B**). Reduced recovery of TRX103 cells was not due to increased killing by PBMC-derived cells, as PBMC-derived CD4^+^ T cells, CD8^+^ T cells, and NK cells all expanded more in the presence of pCD4^ΔIL-10^ controls than with TRX103 (**Figure 3C**; p <0.05).

As predicted by the reduced HLA-DR/DP/DQ expression on TRX103, TRX103 cells exhibited a reduced capacity to induce activation of allogeneic CD4^+^ T cells, as measured in a short-term 24-hour MLR. By measuring co-expression of CD69 and CD137 to assess early activation of allogeneic CD4^+^ T cells, we observed that TRX103 was significantly less likely than pCD4^ΔIL-10^ to stimulate allogeneic T cells (**Figure 3D**; p <0.01). These findings demonstrate a reduced stimulatory capacity by TRX103 cells that limits activation and expansion of allogeneic lymphocytes.

### 3.4 TRX103 modulates immune responses *in vitro*

**Figure 4:**
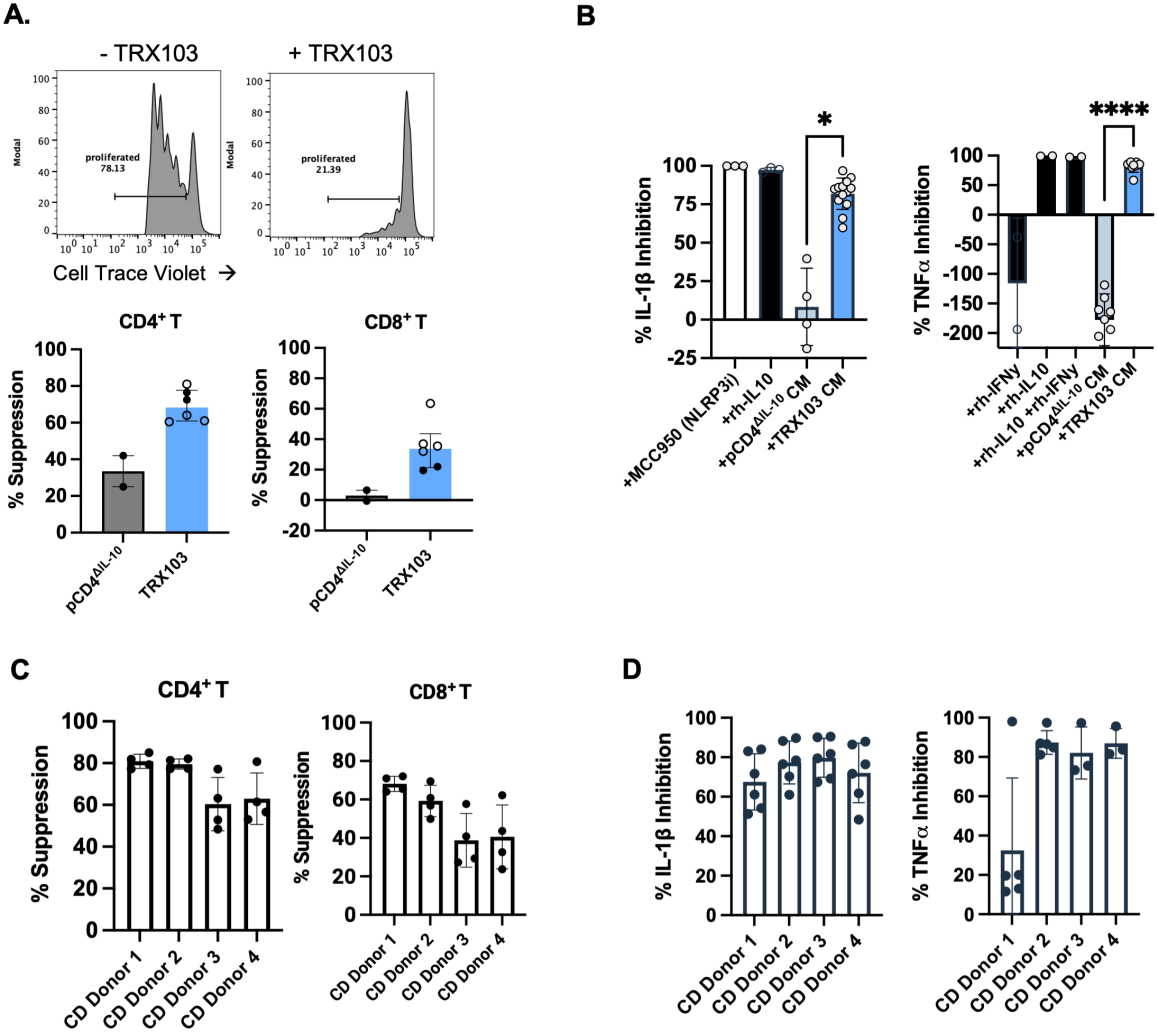
TRX103 modulates both T cell and myeloid cell responses in healthy donors and patients with Crohn’s disease *in vitro*. **(A)** Representative flow cytometry histogram of responder CD4^+^ T cells, with or without TRX103, undergoing cell division as indicated by dilution of Cell Trace Violet. Summarized suppression of CD4^+^ T cells and CD8^+^ T cell proliferation by healthy donor T cells mediated by pCD4^ΔIL-10^ (n = 2) and TRX103 (n = 6). Research-scale pCD4^ΔIL-10^ n = 2 and TRX103 (filled circles, n = 2) and clinical-scale TRX103 (open circles, n = 4). **(B)** Percent inhibition of IL-1β with respect to activated (LPS + Nigericin) monocytes the presence of with by MCC950 (selective small molecule NLRP3 inhibitor), rh-IL10, pCD4^ΔIL-10^ CM (n = 4), or TRX103 CM (n = 13). Percent inhibition of TNF-α with respect to activated (LPS) monocytes in the presence of rh-IFN γ, rh-IL10, both rh-IL10 and rh-IFNγ, pCD4^ΔIL-10^ CM, or TRX103 CM. **(C)** Percent suppression of CD4^+^ T cells and CD8^+^ T cell proliferation from patients with Crohn’s disease mediated by TRX103 CM (n = 4). **(D)** Percent inhibition of IL-1β and TNF-α of activated monocytes from patients with Crohn’s disease mediated by TRX103 CM (n = 3-6). CD = Crohn’s disease; CM = conditioned media; LPS = Lipopolysaccharide; NLRP3i = NLR family pyrin domain containing 3 inhibitor; rh = recombinant human; pCD4^∆IL-10^, pooled non-IL-10 secreting control CD4^∆IL-10^; *p<0.05, ****p<0.0001.

To understand the immunoregulatory capacity of TRX103 cells, we investigated the ability of TRX103 to modulate inflammatory responses and to suppress T cell proliferation *in vitro*. Consistent with previous data for natural Tr1 Tregs, TRX103 specifically suppressed the proliferation of both healthy donor allogeneic CD4^+^ and CD8^+^ T cells *in vitro* (**Figure 4A**), with marked inhibition of T cell division in the presence of TRX103 compared with pCD4^ΔIL-10^ (CD4^+^ T cell: 69% vs 34% suppression; CD8^+^ T cell: 35% vs 3% suppression; n = 2-6). To confirm the central role of secreted IL-10 in the suppression of effector T cells by TRX103, we next evaluated the effect of conditioned media from activated TRX103. As predicted, conditioned media from TCR-activated TRX103 suppressed effector CD3^+^ T cell proliferation, with the degree of suppression reflecting the IL-10 concentrations present in the conditioned media (**Supplementary Figure 4**). The ability of TRX103 to modulate innate immune activation of myeloid cells was also assessed. Monocytes cultured under inflammasome-activating conditions secreted high levels of IL-1β (ranging from 4,960 to 21,899 pg/mL, n = 5) and LPS-activated monocytes secreted high levels of TNF-α (ranging from 1,701 to 3,411 pg/mL n = 2). Coculture with conditioned media (CM) from activated TRX103 cells significantly reduced NLR family pyrin domain containing 3 inflammasome activation, as measured by IL-1β secretion (82% ± 10% inhibition, n = 13), and significantly reduced TNF-α secretion (82% ± 10% inhibition, n = 4; **Figure 4B**). Although TRX103 cells produce IFN-γ (**Supplementary Figure 2B**), a cytokine known to induce TNF-α production in monocytes, TNF-α secretion was inhibited by TRX103, indicating that IL-10–mediated regulation dominates also in the presence of IFN-γ, as previously demonstrated with rh-IL-10 (25). In contrast, CM from the pCD4^ΔIL-10^ control did not suppress inflammatory cytokine secretion by monocytes and, in some instances, increased IL-1β and TNF-α levels. Consistent with an IL-10-dependent mechanism, neutralization of IL-10 or blockade of the IL-10 receptor was sufficient to abrogate the suppressive effects of CM from activated TRX103 (**Supplementary Figure 5**). These findings demonstrated that soluble factors released by TRX103 upon activation suppresses inflammatory cytokine production by myeloid cells.

The immunomodulatory T cell capacity of TRX103 was further confirmed in PBMC isolated from patients with Crohn’s disease. Consistent with the results from healthy donor-derived cells, TRX103 effectively suppressed proliferation of both CD4^+^ and CD8^+^ T cells isolated from Crohn’s disease patients (CD4^+^ T cell: 71% ± 11% suppression, CD8^+^ T cell: 52% ± 14% suppression; n = 4) (**Figure 4C**). In addition, CM from TRX103 significantly reduced IL-1β secretion (74% ± 5% inhibition, n = 6) and TNF-α (72% ± 26% inhibition, n = 3-5) by activated monocytes from Crohn’s disease patients (**Figure 4D**). Thus, TRX103 exerted consistent immunomodulatory effects on monocytes and T cells derived from Crohn’s disease patients.

Together, these data demonstrate that TRX103 exerts immunoregulatory effects on both adaptive and innate immune responses on cells from both healthy donors and Crohn’s disease patients, consistent with the induction of Tr1 Treg cell function by the IL-10 reprogramming.

### 3.5 TRX103 prevents xenogeneic GvHD and protects intestinal tissues *in vivo*

**Figure 5:**
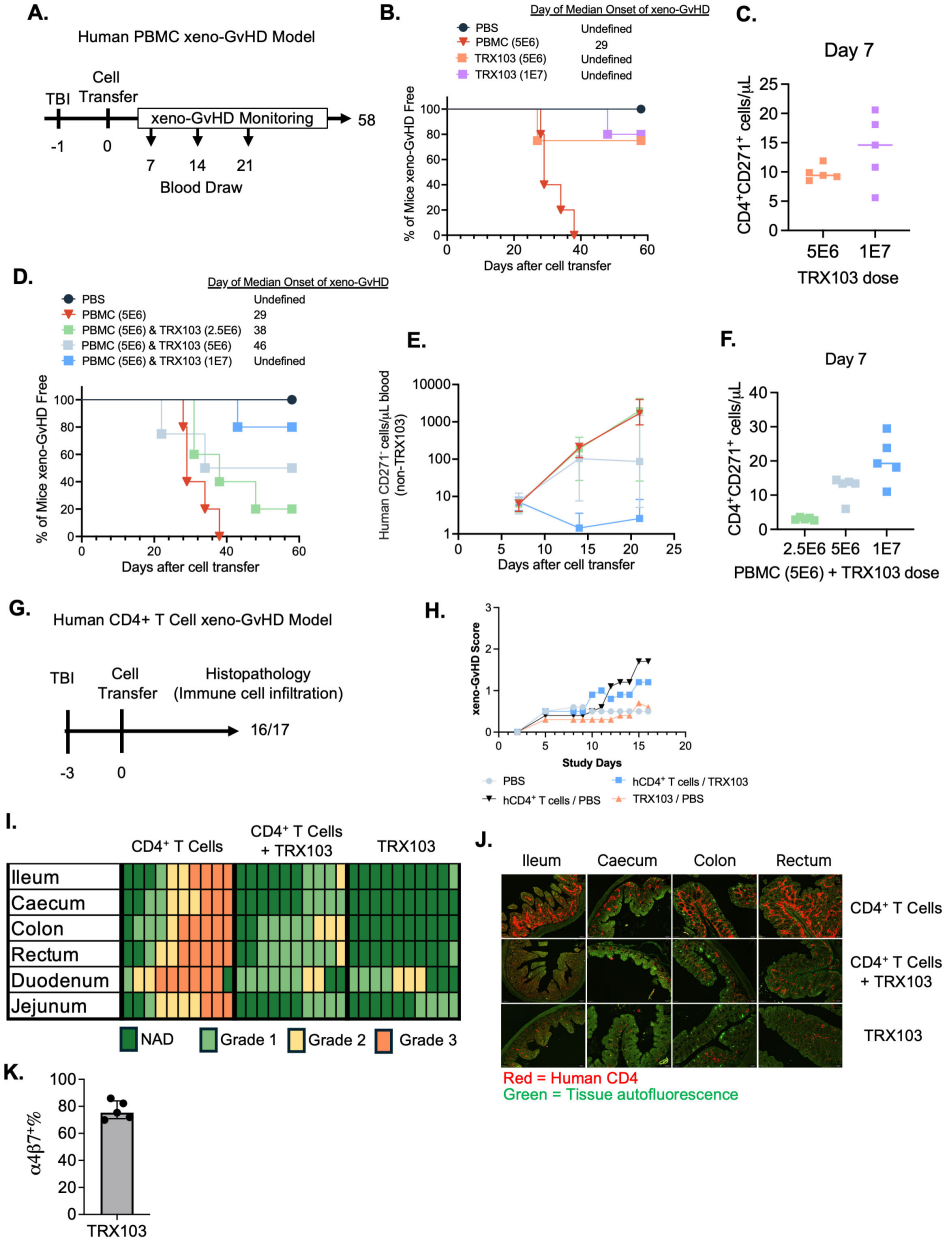
TRX103 has protective functions in a xeno-GvHD NSG humanized mouse model. **(A)** Timeline of PBMC-induced xeno-GvHD mouse model. NSG mice were sub-lethally irradiated and on Day 0 they were intravenously injected with PBS or cells (PBMC alone, TRX103 alone, or in combination). Numbers represent days in study. **(B)** Percentage of mice free from xeno-GvHD are shown for the PBS, PBMC injected alone, or TRX103 injected alone conditions. The median onset of xeno-GvHD is calculated for each group and shown. **(C)** Quantification of TRX103 cells in the blood of mice injected with TRX103 alone after 7 days. **(D)** Percentage of mice free from xeno-GvHD are shown when injected with PBS or with PBMC, with or without TRX103. The median onset of xeno-GvHD is calculated for each group and shown. **(E)** The number of non-TRX103 cells in the blood in the groups with PBMC alone condition or when co-injected with TRX103. **(F)** Quantification of TRX103 cells in the blood of mice co-injected with PBMC and TRX103 after 7 days. **(G)** Timeline of human CD4^+^ T cell-induced xeno-GvHD mouse model. Each cell type is injected as followed: CD4^+^ T cells 5E6 cells/mouse, TRX103 5E6 cells/mouse, or in combination CD4^+^ T cells 5E6 cells/mouse and TRX103 5E6 cells/mouse. Numbers represent days in study. **(H)** xeno-GvHD scores in all groups until termination on Day 16/17. **(I)** Individual immune infiltration scores from microscopic findings per organ. Each box represents a mouse and their immune infiltration score as followed: NAD = Nothing Abnormal Discovered, Grade 1 = minimal, Grade 2= mild, Grade 3 = moderate. **(J)** Immunofluorescence staining of the indicated organs by CD4 (red) across the 3 groups and Autofluorescence (green). **(K)** Frequency of integrin α4β7^+^ cells in TRX103 (n = 5). NSG = NOD.Cg-*Prkdc^scid^ Il2rg^tm1Wjl^*/SzJ, TBI, total body irradiation; xeno-GvHD, xenogeneic graft versus host disease.

The *in vivo* immunoregulatory activity of TRX103, manufactured at clinical scale, was evaluated in a humanized mouse model of GvHD (**Figure 5A**). In this model, adoptive transfer of human PBMC in TBI-NSG mice leads to rapid expansion of these cells and onset of xeno-GvHD within 2–5 weeks, depending on the PBMC donor and cell dose used (26). In this study, adoptive transfer of PBMC led to xeno-GvHD with a median onset and median survival both at 29 days (**Figure 5B**; **Supplementary Figure 6B**). Administration of TRX103 cells in the absence of PBMC at two dose levels (5E6 and 1E7 cells/mouse) did not induce xeno-GvHD within the study duration of 58 days (**Figure 5B**; **Supplementary Figure 6B**). TRX103 cells were detectable in the peripheral blood one-week post-infusion at both dose levels (**Figure 5C**), indicating that lack of xeno-GvHD was not due to a lack of engraftment.

Co-administration of TRX103 with allogeneic PBMC resulted in a dose-responsive delayed onset and reduced incidence of xeno-GvHD with a corresponding increased survival (**Figure 5D**). The median onset of xeno-GvHD in mice injected with PBMC and co-administered TRX103 cells at 2.5E6, 5E6, and 1E7 cells/mouse was 38 days, 46 days, and > 58 days (exceeded the study duration), respectively (**Figure 5D**). TRX103 suppressed the expansion of the PBMC in a dose-specific manner. The highest TRX103 dose of 1E7cells/mouse completely prevented expansion of pathogenic PBMC as quantified by the number of non-TRX103 human cells present in the peripheral blood (**Figure 5E**). On Day 7, the amount of TRX103 cells in the peripheral blood was dose-proportional when co-injected with PBMC (**Figure 5F**). The early dose-response relationship across three dose levels further supports a causal link between TRX103 and disease prevention.

The gastrointestinal tract is a major target organ for GvHD, and preclinical mouse models have demonstrated that adoptive transfer of IL-10 producing Tr1 Tregs protect against colitis induced by transfer of effector CD4^+^ T cells (7, 11). In the PBMC xeno-GvHD model, the majority of the non-TRX103 engrafted human cells are CD4^+^ T cells, which alone are sufficient to induce xeno-GvHD (27). To further define the mode of action of TRX103, we adoptively transferred purified human CD4^+^ T cells to induce xeno-GvHD and specifically assess the impact of TRX103 on xeno-GvHD onset and histopathology. Histopathological analysis was performed on pre-symptomatic mice 16 or 17 days after adoptive cell transfer of 5E6 purified CD4^+^ T cells (**Figure 5G**). At this timepoint, no animals had overt, externally presenting xeno-GvHD symptoms (xeno-GvHD scores <5; **Figure 5H**). However, the subclinical immune infiltration and tissue pathology were already underway in CD4^+^ T cell recipients, and therefore tissue harvest at this early timepoint capture incipient xeno-GvHD-associated damage prior to overt clinical deterioration. Tissue sections across the GI tract were isolated and stained with hematoxylin and eosin and immune infiltration was scored. We observed robust CD4^+^ T-cell infiltration in the gut of mice receiving CD4^+^ T cells which was accompanied by tissue damage (**Figure 5I**). In contrast, co-injection of TRX103 with CD4^+^ T cells substantially reduced both immune infiltration and the associated tissue damage (**Figure 5I**). Importantly, TRX103 alone did not cause any tissue damage. In addition, immunofluorescence staining with anti-CD4 showed that TRX103 indeed homed to the gut and prevented the local expansion of CD4^+^ T cells (**Figure 5J**). As T cell trafficking to gut tissue is associated with expression of integrin α4β7, expression of this molecule was confirmed on TRX103 cells by flow cytometry, consistent with intestinal trafficking potential (**Figure 5K**).

Overall, these data demonstrate that TRX103 confers dose-dependent protection from xeno-GvHD and associated intestinal pathology in this preclinical model, supporting further clinical evaluation as an allogeneic Tr1 Treg-like cell therapy for immune-mediated diseases such as GvHD and Crohn’s Disease.

## 4 Discussion

The development of autologous Tr1 Treg-based cellular therapies has historically been hindered by the biological complexity of the cellular product and by significant manufacturing challenges. Here, we report the preclinical characterization of TRX103, an engineered allogeneic Tr1 Treg-like cell therapy in which enforced IL-10 expression drives a distinct cellular identity — characterized by coordinated downregulation of T cell activation, antigen presentation, and adhesion pathways — that translates to reduced proliferation and allogeneic stimulatory capacity alongside potent suppression of T cell and myeloid inflammatory responses *in vitro*. *In vivo*, TRX103 prevented xeno-GvHD in a dose-dependent manner, homed to intestinal tissues, and protected against CD4^+^ T cell-driven immune infiltration and tissue damage. Together, these findings establish TRX103 as a scalable and potent off-the-shelf Tr1 Treg-like cell therapy with a preclinical profile and biological mechanism of action supporting its clinical evaluation in both prevention of GvHD and treatment of Crohn’s disease.

The molecular basis of TRX103’s functional profile is rooted in the effects of enforced IL-10 expression. IL-10 overexpression led to a selective remodeling of the cell surface, with predominant downregulation of adhesion, antigen presentation, and activation markers. CD49f, which encodes integrin α6 protein and binds to laminin (28), which is rich in basement membranes, was confirmed to be significantly upregulated in IL-10-transduced cells compared to pCD4^ΔIL-10^ controls. It has been reported that CD49f^+^ FOXP3^+^ Tregs from healthy donors express higher IL-10 than their CD49f^−^ counterparts (29), consistent with a linkage between IL-10 signaling and CD49f expression. Whether CD49f upregulation on TRX103 reflects autocrine IL-10/STAT3 signaling or an indirect consequence of IL-10-mediated signaling warrants further investigation.

When the surface phenotype of TRX103 is compared with that of naturally occurring Tr1 Tregs, key differences emerge, likely reflecting *in vivo* terminal differentiation of naturally occurring Tr1 Tregs. The surface markers of natural Tr1 Tregs include LAG-3, CD49b, and CD226 (7); however differential upregulation of these molecules was not observed in TRX103 relative to pCD4^ΔIL-10^ controls. LAG-3 and CD49b are broadly expressed across our ex vivo-activated and expanded T cells regardless of IL-10 transduction status, and their expression in TRX103 is consistent with this general pattern of ex vivo culture-induced expression. Thus, their lack of differential upregulation relative to controls likely reflects the ubiquity of these markers under culture conditions rather than an inability to adopt Tr1 Tregs identity. Together, while TRX103 shares core functional attributes with natural Tr1 Tregs, its surface phenotype reflects both the influence of IL-10 signaling and the specific conditions of its manufacturing process.

Our study advances the development of Tr1 Treg-based cellular therapies by demonstrating that a pooled, allogeneic, IL-10-engineered Tr1 Tregs product can be manufactured reproducibly at a scale to support broad clinical use. The process implements several key improvements over previous methods (20, 22), replacing feeder-dependent expansion with a GMP-compliant synthetic TCR activator, and using cryopreserved CD4^+^ T cell starting material, which extends the donor collection window and enables extensive upfront characterization prior to manufacturing initiation. Critically, pooling three sublots enables production of larger commercial batch quantities. In the context of advanced cell therapies — where lot release is exceptionally rigorous, costly, and consumes a significant proportion of the final product — producing larger lots reduces the fraction consumed for quality control per-batch testing and allows each lot to treat more patients. Furthermore, this pooling strategy minimizes lot-to-lot variability as a confounding variable, allowing for clearer interpretation of therapeutic activity across diverse clinical indications.

A major hallmark of TRX103 is its significantly reduced allogeneic stimulatory capacity, which would predict a lower likelihood of rejection by the patient’s immune system. Specifically, TRX103 exhibits markedly reduced expression of critical adhesion, activation, and antigen-presentation molecules, including CD54, CD69, CD80, CD86, and HLA-DR/DP/DQ. This orchestrated downregulation is highly desirable for an off-the-shelf cell therapy because it limits allogeneic stimulation and off-target inflammation, effectively reducing the risk of allogeneic rejection without requiring complex genomic engineering. In contrast to TRX103, current immune evasion strategies for allogeneic cell therapy typically rely on disrupting HLA Class I through beta-2 microglobulin knockout and overexpressing inhibitory ligands such as HLA-E, CD47, or PD-L1 to prevent NK cell-mediated rejection, which introduces manufacturing complexity, potential safety concerns, and regulatory challenges (30). TRX103 achieves a comparable reduction in allostimulatory capacity through a single transgene, IL10, which simultaneously delivers therapeutic function. While IL-10 has previously been shown to downregulate HLA Class II and CD80/CD86 on antigen-presenting cells, here we establish this effect in the T cells that compose the TRX103 product, indicating that enforced IL-10 expression may enable a streamlined approach to reducing allogeneic immunogenicity without multi-gene editing. This dual-purpose mechanism simplifies product design while retaining intact HLA Class I expression, which protects TRX103 from NK cell-mediated killing without the need for additional engineering. However, while the reduced HLA Class II and costimulatory molecule expression observed here strongly supports a lower risk of allogeneic rejection, the possibility of CD8^+^ T cell-mediated recognition and elimination of TRX103 via intact HLA Class I, as well as humoral immune responses upon repeated dosing, remain important open questions that will require evaluation in immune-competent recipients.

TRX103 is currently undergoing clinical evaluation in two Phase 1/2a trials: for prevention of GvHD (NCT06462365) and in treatment-refractory Crohn’s disease (NCT06721962). These disease indications represent distinct and complementary settings for evaluation of Tr1 Treg-based therapeutics, offering a unique opportunity to understand how Tr1 Treg biology influences therapeutic performance across different immune-mediated diseases. In HSCT, patients undergo conditioning regimens that profoundly deplete the immune system prior to donor cell engraftment. Here, the primary goal of TRX103 is to control pathogenic alloreactivity during immune reconstitution without triggering additional immune activation (31). Consistent with this, TRX103 showed minimal immunostimulatory activity *in vitro*, did not induce xeno-GvHD when administered alone in immunocompromised mice, and effectively prevented disease in a xeno-GvHD model, supporting its potential to prevent GvHD in the clinic. While the essential role of IL-10 for disease protection in this model has been established by prior studies using research-scale cell products (20, 21), additional regulatory pathways may also contribute, although these were not explicitly tested in this *in vivo* system. It should be noted, however, that these data address GvHD prevention— an important but inherently limited framing, given that not all HSCT recipients will develop GvHD and that the ability of TRX103 to reverse established acute GvHD or provide benefit in chronic GvHD arising long after transplant remains an open and important clinical question.

By contrast, treatment with TRX103 in Crohn’s disease occurs in patients with ongoing disease and a dysregulated immune system. In addition, it is well established that this patient population is marked by heterogeneity in disease duration, location, severity, and inflammatory pathways (32). Regulatory cell therapies in this setting must selectively dampen pathological inflammation while preserving overall host immunity. Unlike T cell therapies that rely on synthetic T cell receptors or chimeric antigen receptors to drive antigen-specific activation, TRX103 retains an intact, polyclonal T cell receptor repertoire, enabling broad responsiveness to diverse inflammatory cues — an attribute that may be particularly advantageous in a disease with heterogeneous and incompletely characterized antigenic drivers such as Crohn’s. TRX103’s expression of gut-homing molecules and potential for robust local secretion of IL-10 further suggests its ability to traffic to inflamed intestinal tissues, locally modulate pathological processes, and possibly restore immune homeostasis.

The development of TRX103 occurs within a rapidly advancing landscape of regulatory T cell therapies for immune-mediated diseases. In the GvHD setting, adoptive transfer of ex vivo isolated and ex vivo expanded FOXP3^+^ Tregs has demonstrated safety and potential clinical benefit (33, 34). In IBD, early-phase clinical studies of autologous, expanded FOXP3^+^ Tregs have reported safety and preliminary evidence of clinical benefit in ulcerative colitis (35). Early indicators of efficacy in IBD for both FOXP3^+^ Tregs and Tr1 Tregs illustrate their overlapping suppressive functions in some contexts — including inhibition of T cell proliferation and modulation of myeloid cell activation. The homeostatic roles of FOXP3^+^ Tregs and Tr1 Tregs are complementary rather than redundant, as FOXP3^+^ Tregs rely predominantly on contact-dependent suppression and IL-2 consumption, whereas Tr1 Tregs act primarily through cytokine-mediated suppression, particularly via IL-10 (2). Additionally, IL-10 secretion by Tr1 Tregs enables suppression of NLRP3 inflammasome activation in myeloid cells — a mechanism of immunoregulation not well-described for FOXP3^+^ Tregs. While FOXP3^+^ Tregs include tissue-resident populations that maintain local homeostasis (36), TRX103 cells express gut-homing integrins such as α4β7, suggesting the potential to preferentially deliver IL-10 within the intestinal mucosal microenvironment — an attribute relevant to both Crohn’s disease and GvHD, in which the gut is a primary target organ. This mechanistic distinction, combined with differences in gut-homing capacity and the inflammatory microenvironment of each disease, suggests that certain indications may be inherently more responsive to one regulatory strategy over the other (37).

In conclusion, TRX103 addresses longstanding challenges in Tr1 Treg cell therapy by combining a scalable, GMP-compatible manufacturing process with a functionally potent, IL-10-dominant immunoregulatory phenotype that is mechanistically suited to diseases driven by pathogenic T cell and myeloid activation. The dual-purpose nature of the IL-10 transgene, simultaneously conferring therapeutic function and reducing allogeneic immunogenicity through a single genetic modification, distinguishes TRX103 from both conventional Treg approaches and more heavily engineered allogeneic cell therapies. While the present study is limited to preclinical characterization, ongoing Phase 1/2a trials in GvHD prevention (NCT06462365) and treatment-refractory Crohn’s disease (NCT06721962) will provide the first clinical data on TRX103’s safety, persistence, and efficacy in immune-competent recipients.

Together, this work advances the scientific foundation for IL-10-based regulatory cell therapy and establishes a framework for clinical evaluation across T cell- and myeloid-driven immune-mediated diseases.

## Data Availability

The original contributions presented in the study are included in the article/**Supplementary Material**. Further inquiries can be directed to the corresponding author.
