# Supplementary material for: Engineering allogeneic type 1 regulatory T cells: a scalable, off-the-shelf platform for restoring immune tolerance
Source: Front Immunol. 2026 Jul 9;17:1848770. doi: 10.3389/fimmu.2026.1848770 (PMC13391293; doi:10.3389/fimmu.2026.1848770)
Supplement: Supplementary file 1 [file Supplementaryfile1.docx]

Supplementary Material

# Supplementary Figures and Tables

## Table S1: Antibodies and Reagents

| Target | Fluorochrome | Company | Catalog | Clone |
| --- | --- | --- | --- | --- |
| CD8 | APC | BioLegend | 344722 | SK1 |
| CD4 | FITC | BioLegend | 300506 | RPA-T4 |
| CD3 | Pacific Blue | BioLegend | 300431 | UCHT1 |
| CD271 (NGFR) | PE/Cy7 | BioLegend | 345110 | ME20.4 |
| CD45 | APC/Cy7 | BioLegend | 304014 | HI30 |
| CD4 | PerCP-Cy5.5 | BioLegend | 300530 | RPA-T4 |
| CD19 | Brilliant Violet 605 | BioLegend | 302244 | HIB19 |
| CD14 | FITC | BioLegend | 325604 | HCD14 |
| CD56 (NCAM) | PE | BioLegend | 318306 | HCD56 |
| CD16 | PE | BioLegend | 302008 | 3G8 |
| CD4 | Brilliant Violet 650 | BioLegend | 317436 | OKT4 |
| CD3 | PerCP-Cy5.5 | BioLegend | 344808 | SK7 |
| CD56 (NCAM) | PE | BioLegend | 362524 | 5.1H11 |
| CD19 | PE | BioLegend | 302254 | HIB19 |
| CD14 | PE | BioLegend | 301850 | M5E2 |
| CD8 | APC/Cy7 | BioLegend | 344714 | SK1 |
| CD69 | FITC | BioLegend | 310904 | FN50 |
| CD137 | AlexaFluor 647 | BioLegend | 309824 | 4B4-1 |
| IL-2 | Brilliant Violet 421 | BioLegend | 500328 | MQ1-17H12 |
| IFNɣ | Brilliant Violet 786 | BD Biosciences | 563731 | 4S.B3 |
| HLA-A2 | Brilliant Violet 510 | BioLegend | 343320 | BB7.2 |
| Zombie Aqua Fixable Viability Kit | N/A | BioLegend | 423102 | N/A |
| Zombie R718 Fixable Viability Kit | N/A | BioLegend | 423116 | N/A |
| CD45 | Brilliant Violet 785 | BioLegend | 304048 | HI30 |
| CD45 (mouse) | Pacific Blue | BioLegend | 30-F11 | 103126 |
| CD3 | FITC | BioLegend | 317306 | OKT3 |
| CD4 | Spark YG593 | BioLegend | 344672 | SK3 |
| CD8 | Brilliant Violet 605 | BioLegend | 344742 | SK1 |
| CD271 | Alexa Fluor 647 | BioLegend | 345114 | ME20.4 |
| CD45 | VioBlue | Miltenyi Biotec | 130-110-637 | REA747 |
| CD4 | PE-Cy7 | BioLegend | 300512 | RPA-T4 |
| CD271 | Brilliant Violet 711 | BD Biosciences | 743360 | C40-1457 |
| α4β7 | PE | R&D Systems | FAB10078P | Vedolizumab Biosimilar |
| Human IL-10 Secretion Assay- Detection Kit (PE), Miltenyi Biotech (100 tests) | PE | Miltenyi Biotec | 130-091-376 | N/A |
| Viability | Fixable Viability Dye eFluor 780 | Invitrogen eBioscience | 65-0865-14 | N/A |

## Table S2: Xenogeneic (Xeno) Graft Versus Host Disease Scoring

| **Score** | **0** | **1** | **2** |
| --- | --- | --- | --- |
| **Weight Loss** | ≤ 10% loss | 10–25% | > 25% |
|  | < 10% loss | ≥10%, < 20% | ≥ 20% |
| **Activity** | Normal | Mild to moderately decreased | Stationary unless stimulated |
| **Posture** | Normal | Hunching only at rest | Severe hunching impairs movement |
| **Fur** | Normal | Mild to moderate ruffling | Severe ruffling / poor grooming |
| **Skin Integrity** | Normal | Scaling of paws / tail | Obvious areas of denuded skin |

Each of the five categories are scored from 0 – 2 and the scores of each category are combined (maximum score of 10). Mice with a combined score ≥ 7 and / or a weight loss ≥ 30% were euthanized for humane reasons. Two different criteria for weight loss scoring were used depending on the facility and model. In the top row, the weight loss criteria were used for the PBMC induced xeno-GvHD model conducted in the USA. In the bottom row, the weight loss criteria were used for the CD4^+^ T cell induced xeno-GvHD model conducted in Italy.

## Supplementary Figures

**Supplementary Figure 1.** Pooling 3 CD4^IL-10^ sublots increases total TCR clonotype richness in product. Two lots of TRX103 and their respective CD4IL-10 sublots were TCR-sequenced. All CD4^+^ T cell donors used were unique and not shared across TRX103 lots. The top lot was manufactured at clinical-scale in the CliniMACS Prodigy and the bottom lot was manufactured at research-scale in G-Rex vessels. The number of unique clonotypes that make up the top 50% are indicated below each graph. Each bar represents a different clonotype, with some colors repeating due to the limited number of colors available. CD4^IL-10^ sublot 4.1— V5-J22 (black bar; 11.25%), V5-J11 (light grey bar; 6.71%), and V24-J23 (blue bar; 6.65%). TRX103 product – V5-J22 (black bar; 7.41%), V5-J11 (light grey bar; 3.60%), and V24-J23 (blue bar; 3.45%).

**Supplementary Figure 2. Verification of a Tr1 Treg cytokine profile in cell culture supernatants containing TRX103 after 48 hours.** Cytokine-containing supernatants were assessed by ELISA. **(A)** Cytokine quantification of cell culture supernatants containing vector-control (pCD4^ΔIL-10^, n = 3). pCD4^ΔIL-10^ were generated using the same process as TRX103 but were transduced with an LVV encoding a mutated, non-functional IL-10 transgene. The ΔIL-10 transgene prevents IL-10 translation. One value was below the limit of detection and was set to the limit of detection (0.031 ng/mL). (**B)** Cytokine quantification of TRX103 cell culture supernatants (n = 4). Unstimulated = media only, Stimulated = ImmunoCult T Cell Activator (anti-CD3/CD28/CD2, Stem Cell Technologies). The mean ± standard deviation is plotted for each respective cytokine in ng/mL.

**Supplementary Figure 3. Expression of LEGENDScreen hits on TRX103 and controls.** Extended flow validation summarized on LEGENDScreen hits on additional TRX103 lots (blue: research-scale, n = 5; red: clinical-scale, n = 4) and respective controls (grey, n = 5). Values plotted are the frequency of positive cells of the indicated marker within the CD4^+^CD271^+^ cell population. pCD4^∆IL-10^ = pooled non-IL-10 secreting control CD4^∆IL-10^. *p<0.05, ***p<0.001.

**Supplementary Figure 4. TRX103 secretes soluble factors capable of suppressing effector T cell proliferation.** Conditioned media were generated from TRX103 lots 6 and 9 by stimulating cells with Human T-Activator CD3/CD28 Dynabeads (Thermo Fisher Scientific) at a 1:1 bead-to-cell ratio for 72 hours, after which cell culture supernatants were harvested. CellTrace Violet (CTV)-labeled effector CD3⁺ T cells were co-cultured with monocytes at a 10:1 ratio (T cell: monocyte) in the presence of soluble anti-CD3 antibody and either 2.5% (v/v) TRX103-conditioned media (corresponding to 1.18 ng/mL and 1.74 ng/mL of IL-10 for lots 6 and 9, respectively) or recombinant human IL-10 (rh-IL10) at the indicated concentrations for 96 hours. Effector CD3⁺ T cell proliferation was assessed by CTV dye dilution via flow cytometry. Percent suppression was calculated as (proliferation of responders alone - proliferation of responders with suppressors)/proliferation of responders alone.

**A**

**B**

**Supplementary Figure 5. TRX103-conditioned media suppresses IL-1β and TNF-α production by activated monocytes via IL-10/IL-10R signaling.** Frozen monocytes from two healthy donors (Monocyte Donor 178 and Monocyte Donor 181) were thawed and stimulated with LPS + Nigericin in the presence of 25% (v/v) TRX103-conditioned media (CM) recombinant human (rh) IL-10 (10 ng/mL), or neutralizing antibodies targeting the IL-10 receptor (anti-IL-10R; clone 3F9, 30 µg/mL; BioLegend) or IL-10 (anti-IL-10; clone JES3-9D7, 30 µg/mL; BioLegend), each alone or in combination with CM. Cell culture supernatants were harvested after 24 hours and secreted levels of **(A)** IL-1β and **(B)** TNF-α (pg/mL) were measured. Values above bars indicate the average concentrations for each condition; dots represent individual TRX103 CM lots where applicable (n=3 independent TRX103 lots). Grey bars = no blocking antibodies; Blue bars = anti-IL-10R conditions; green bars = anti-IL-10 conditions.

**Supplementary Figure 6. Survival curves and immunophenotype strategy in PBMC-induced xeno-GvHD Model. (A)** Immunophenotyping gating strategy. The non-TRX103 human CD45^+^ cells count is calculated by subtracting the CD4^+^CD271^+^ event count from the total human CD45^+^ event count. **(B)** Kaplan-Meier curves of the overall survival regardless of xeno-GvHD status. An “Undefined” day of median survival is generated when the median survival date exceeds the study duration of 58 days**.**
